# 

**Published:** 1891-09

**Authors:** 


### CONTENTS FOR SEPTEMBER, 1891.

ORIGINAL COMMUNICATIONS.

Some After-Effects of the Recent Influenza. By Frank Hamilton Potter, M. D. .......... 65
Duties of Physicians as Examiners for Life Insurance. By George L. Brown, M. D. ........ 69
Concentrated Food in the Treatment of Pulmonary Consumption. By Thomas J. Mays, M. D. .......... 74

SOCIETY PROCEEDINGS.

Philadelphia County Medical Society. .......... 77
Jefferson County [Alabama] Medical Society .......... 89
Buffalo Medical and Surgical Association .......... 94
The Medical Society of Chautauqua County .......... 96

PROGRESS IN MEDICAL SCIENCE.

Physiological Chemistry .......... 97
Medico-Legal Notes .......... 99
Ophthalmology .......... 102
Medicine .......... 104

SELECTION.

Somnal—A Hypnotic .......... 105

EDITORIAL.

Koch vs. Virchow .......... 108
Important to Medical Students .......... 109
Membership in the American Medical Association .......... 109
Correction .......... 110

PERSONAL.

Dr. George H. Rohé—Dr. L. Duncan Bulkley—Prof. John A. Miller—Dr. F. B. Darling—Dr. Russell Bellamy—H. M. Whelpley—Dr. E. E. Montgomery—Dr. John H. Rauch—Gen. James A. Beaver .......... 111

SOCIETY MEETINGS.

The American Public Health Association .......... 111
The Mississippi Valley Medical Association .......... 112
The Third Annual Meeting of the Tri-State Medical Association .......... 112
The American Association of Obstetricians and Gynecologists .......... 112
The Canadian Medical Association—The American Orthopedic Association—The American Electro-Therapeutic Association .......... 113

OBITUARY.

Dr. James W. Craig .......... 113

JOURNALISTIC NOTES.

A Magazine Written by Women .......... 114
*New England Medical Monthly*—The *Climatologist*—*Anales del Departaments Nacional de Higiene* .......... 115

MEDICAL COLLEGE NOTES.

College Openings .......... 115
Tulane University—Reorganization of the Chicago College of Physicians and Surgeons... 116
Medico-Chirurgical College of Philadelphia—Long Island College Hospital .......... 117

BOOK REVIEWS.

A Manual of the Practice of Medicine .......... 117
Practical Points in the Management of Some of the Diseases of Children .......... 118
Fever: Its Pathology and Treatment by Antipyretics .......... 119
Origin, Purpose and Destiny of Man—Transactions of the American Gynecological Society .......... 120
The Pocket Anatomist—Transactions of the Association of American Physicians .......... 121
Plain Talks on Electricity and Batteries—The Action, Therapeutic Value and Use of the Carlsbad Sprudel Salt .......... 122
A Guide to the Practical Examination of the Urine .......... 123
Books and Pamphlets Received .......... 124

